# Phenotypic and Functional Properties of Tumor-Infiltrating Regulatory T Cells

**DOI:** 10.1155/2017/5458178

**Published:** 2017-12-31

**Authors:** Gap Ryol Lee

**Affiliations:** Department of Life Science, Sogang University, 35 Baekbeom-ro, Mapo-gu, Seoul 04107, Republic of Korea

## Abstract

Regulatory T (Treg) cells maintain immune homeostasis by suppressing excessive immune responses. Treg cells induce tolerance against self- and foreign antigens, thus preventing autoimmunity, allergy, graft rejection, and fetus rejection during pregnancy. However, Treg cells also infiltrate into tumors and inhibit antitumor immune responses, thus inhibiting anticancer therapy. Depleting whole Treg cell populations in the body to enhance anticancer treatments will produce deleterious autoimmune diseases. Therefore, understanding the precise nature of tumor-infiltrating Treg cells is essential for effectively targeting Treg cells in tumors. This review summarizes recent results relating to Treg cells in the tumor microenvironment, with particular emphasis on their accumulation, phenotypic, and functional properties, and targeting to enhance the efficacy of anticancer treatment.

## 1. Introduction

Regulatory T (Treg) cells are CD4 T cells that inhibit immune responses. Treg cells express high amounts of CD25 and transcription factor Forkhead box protein 3 (Foxp3) [1, 2]. Treg cells maintain immune homeostasis by inhibiting immune responses. These cells not only protect tissues from excessive immune responses but also suppress immune responses against self-antigens, innocuous environmental antigens, antigens from food and microbiota, and fetal antigens during pregnancy.

Treg cells inhibit immune responses by a variety of mechanisms, including the secretion of anti-inflammatory cytokines such as interleukin- (IL-) 10, tumor growth factor- (TGF-) *β*, and IL-35 [3]. In addition, Treg cells express high levels of IL-2R, depleting IL-2, a growth factor for effector T (Teff) cells, in the surrounding environment. Treg cells also kill Teff cells directly through the FasL-Fas pathway as well as through granzyme-/perforin-mediated cytotoxicity, disrupting the metabolism of Teff cells. Moreover, Treg cells can suppress immune responses by inducing tolerogenic dendritic cells (DCs) [3].

Two types of Treg cells have been identified. Thymus-derived Treg (tTreg) cells develop in the thymus, whereas periphery-derived Treg (pTreg) cells differentiate from naive CD4 T cells in the periphery.

Incipient tumor cells are removed by immune system cells; specifically, CD8^+^ cytotoxic T lymphocytes (CTLs) kill tumor cells, aided by CD4^+^ T cells. Tumor cells express tumor-associated antigens (TAAs), which are newly expressed or mutated self-antigens, and are recognized and killed by CTLs, a phenomenon known as “cancer immune surveillance.” Continual generation of cancer cells and removal by immune cells can be balanced and can last for a long time. Some of these cancer cells may eventually evade immune responses and grow unchecked. Thus, immune system cells are critical in keeping cancers under control.

**Figure 1:**
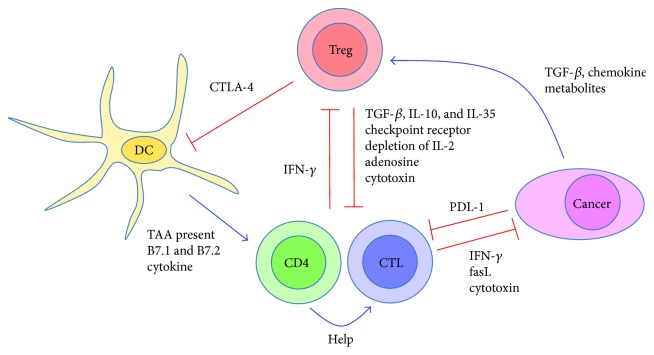
The role of TI-Treg cells in TME. A schematic illustration of the role of TI-Treg cells in the TME. Activation is shown as blue arrows, and inhibition is shown as red blocked lines. TI-Treg cells inhibit CTLs and CD4 Teff cells by secreting anti-inflammatory cytokines, expressing checkpoint receptors, disturbing metabolism, and killing directly. TI-Treg cells also intercept costimulatory signal on DCs by CTLA-4, preventing activation of Teff cells. Cancer cells attract Treg cells to tumor by secreting chemokines and nurture Treg cells by secreting TGF-*β* and immunosuppressive metabolites.

Treg cells infiltrate tumors and inhibit antitumor immune responses by tumor antigen-specific CD8 T cells and CD4 T cells. Thus, Treg cells can block cancer immunotherapy. Because depleting Treg cells throughout the entire body cause fulminant autoimmunity, targeting tumor-infiltrating Treg (TI-Treg) cells can enhance tumor immunotherapy without inducing deleterious autoimmune diseases. Understanding the properties of TI-Treg cells and their methods of suppressing anticancer treatment is essential to achieve this goal. This review summarizes recent findings of TI-Treg cell properties and their therapeutic application (summarized in Figure 1).

## 2. TI-Treg Cells

Cancer cells accumulate mutations during tumorigenesis and acquire the ability to establish their own protective environment, called the tumor microenvironment (TME). The TME contains many types of cells, including cancer cells, immune system cells, fibroblasts, pericytes, and occasionally adipocytes [4, 5]. The immune cells in the TME include CD8 T cells, CD4 T cells, Treg cells, DCs, macrophages, natural killer cells, B cells, and mast cells [4, 5]. These cells establish an environment that is highly immunosuppressive, tolerogenic, hypoxic, and rich in proangiogenic factors. Because Treg cells have immunosuppressive properties, Treg cells in the TME are generally thought to inhibit antitumor activity mediated by Teff cells and to promote tumor growth [6]. Secreted and/or surface molecules in the TME influence the growth of cancer cells. Immunosuppressive cytokines, such as TGF-*β* and IL-10, inhibit antitumor immunity mediated by Teff cells and boost the activity of Treg cells.

High numbers of Treg cells and low CD8 T cell to Treg cell ratios have been found to correlate with poor prognosis and reduced survival of patients with many types of cancer, including ovarian cancer [7, 8], lung cancer [9], pancreatic ductal adenocarcinoma [10, 11], non-Hodgkin's lymphoma [12], glioblastoma [13], melanoma, and other malignancies [14, 15]. By contrast, high numbers of Treg cells were found to correlate with good prognosis in patients with colorectal [16], head and neck [17], and gastric [18] cancer. One explanation of this discrepancy is that Treg cells that reduce inflammation may inhibit the growth of certain types of cancer that depend heavily on inflammation [19]. Inflammation has been shown to contribute to cancer initiation and progression, neoplastic transformation, and metastasis [20]. Alternative explanation is that the discrepancy is caused by inability to quantify heterogeneous Treg cell subsets or the concomitant inflammation in the tumors [21]. Treg cell heterogeneity has been proven in colorectal cancer [22].

## 3. Recruitment and Expansion of Treg Cells in the TME

Increases in the numbers of Treg cells in the TME may result from the preferential recruitment of TI-Treg cells over conventional T (Tconv) cells, increased Treg cell proliferation, and/or conversion of Tconv cells to Treg cells.

### 3.1. Treg Cell Recruitment into the TME

Preferential recruitment of Treg cells into the TME may result from interactions between chemokines and their receptors. Chemokines produced by tumors, including CC chemokine ligand 22 (CCL22), CCL17, CXC chemokine ligand 12 (CXCL12), and CCL28, recruit Treg cells into tumors [23]. Cancer cell-produced CCL22 or CCL17 attracts CC chemokine receptor 4-positive (CCR4^+^) Treg cells in the TME, which seems to be the most prevalent mechanism for Treg cell migration to tumors [7, 24]. Blocking CCR4 reduces the number of intratumoral Treg cells and enhances antitumor immunity [25, 26]. The CCL5/CCR5 axis also plays a role in Treg cell recruitment [27], and hypoxia-induced CCL28 has been found to attract CCR10^+^ Treg cells into ovarian cancers [28].

### 3.2. Expansion of Treg Cells in the TME

TI-Treg cells exhibit increased proliferation, as evidenced by high expression of Ki-67, compared with Treg cells from peripheral blood and healthy tissue [29]. This increased proliferation of TI-Treg cells may be related to their recognition of self-antigens and the nurturing environment in the TME. Higher numbers of prostate-specific Treg cells accumulate in the prostate than in other organs, suggesting that the presence of self-antigens may trigger the expansion of Treg cells in tumors [30]. TI-Treg cells show high surface expression of CD25 (high-affinity IL-2 receptor subunit *α*), allowing these cells to absorb available IL-2 in the environment. This results in the high proliferation of Treg cells but inhibits the growth of Tconv cells in the TME.

Recent evidence shows that metabolic fitness is associated with the preferential expansion of TI-Treg cells in the TME [31]. Because cancer cells preferentially acquire energy from glycolysis, the TME is rich in immunosuppressive metabolites [32–35]. These conditions suppress Teff cell function, while enhancing the function of Treg cells. The differentiation and function of Treg cells preferentially involve fatty acid oxidation and oxidative phosphorylation [36–38], as well as the uptake of lactic acid from the surrounding environment [39, 40], resulting in the metabolic fitness of TI-Treg cells. Several fatty acid-binding proteins are specifically expressed in TI-Treg cells in breast cancers, but not in Treg cells in peripheral blood and normal tissues [41]. It remains unclear, however, whether these metabolites contribute to the expansion of TI-Treg cells.

### 3.3. Conversion of Tconv Cells into Treg Cells

The TME is rich in immunosuppressive molecules, including TGF-*β*, IL-10, and VEGF, suggesting that Tconv cells are converted to Treg cells through the formation of tolerogenic antigen-presenting cells (APCs) in the TME [42–44]. Indoleamine2,3-dioxygenase- (IDO-) expressing APCs may induce the conversion of Tconv to Treg cells through an aryl hydrocarbon receptor [45]. Myeloid-derived suppressor cells (MDSCs) in the TME may also promote the differentiation of Treg cells in an IDO-dependent manner.

It remains unclear, however, whether Tconv cells can be converted to Treg cells in TME. In mouse tumor model, injection of MCA-38 colon adenocarcinoma cells causes enrichment of neuropilin-1- (Nrp1-) pTreg cells, whereas that of 4T1 breast cancer cells causes enrichment of Nrp1^+^ tTreg cells, suggesting that both tTreg and pTreg cells can be enriched in the TME depending on the types of tumor [46]. Analyses showed that TI-Treg cells and Tconv cells have a largely nonoverlapping T cell receptor (TCR) repertoire and that TI-Treg cells originate from tissue-specific Treg cells generated in the thymus [30, 47, 48], suggesting that conversion did not occur. Further studies are needed to resolve this issue.

## 4. Phenotypes and Suppressive Mechanisms of TI-Treg Cells

### 4.1. Phenotypes of TI-Treg Cells

TI-Treg cells exhibit more highly activated phenotypes than Treg cells in the peripheral blood and healthy tissue [49]. TI-Treg cells express high amounts of distinct markers, including CD25, cytotoxic T lymphocyte-associated protein 4 (CTLA-4), glucocorticoid-induced tumor necrosis factor receptor family-related genes (GITR), programmed death-1 (PD-1), lymphocyte activation gene-3 (LAG-3), T cell immunoglobulin and mucin domain-containing-3 (TIM-3/HAVCR2), and inducible T cell costimulator (ICOS). These cells are CD44^high^, CD62L^low^, and CCR7^low^, indicating an effector-memory phenotype [50]. TI-Treg cells have greater immunosuppressive activity than other Treg cells, possibly due to stimulation by TAAs. TAAs originate from self-antigens and bind more strongly by Treg cells than by Teff cells, as Treg cells have higher affinity TCR than Teff cells, leading to preferential activation of Treg cells.

The stability and suppressive function of TI-Treg are very important in tumor growth. Several factors were recently shown to be important in maintaining the stability and suppressive activity of TI-Treg cells, either positively or negatively. The stability of TI-Treg cells and their ability to potentiate immunosuppressive functions were shown to involve the Sema4a-Nrp1 pathway, specifically in tumors but not in other tissues [51]. Treg-specific deletion of Nrp1 was found to block tumor growth in several animal models of cancer [51]. Molecularly, the Sema4a-Nrp1 interaction inhibits Akt phosphorylation by phosphatase and tensin homologue (PTEN), resulting in the nuclear localization of Foxo3a [51]. PI3K is also important for the suppressive activity of TI-Treg cells. CD8 T cell activity was enhanced, and tumor burden was reduced in Treg-specific PI3K p110*δ*-deficient mice [52]. Foxo1 was also found to be important in regulating the generation of activated Treg cells in the TME. Treg-specific Akt-insensitive mutant mice, in which Foxo1 is not repressed, show strong antitumor activity due to lack of activated Treg cells, along with a concomitant increase in intratumoral CD8 T cells [53]. NF-*κ*B c-Rel are also important in the suppressive activity of TI-Treg cells. Treg-specific deletion of c-Rel reduces the expression of activated Treg-specific marker genes, including *Itgae*, *Tigit*, *Klrg1*, *Il1r2*, and *Tnfsf8*, as well as inhibiting tumor growth in the B16F1 melanoma transplantation model; however, these cells do not show an overt autoimmune phenotype [54]. Helios was also shown to be important in TI-Treg cell stability and suppressive activity [55]. Treg-specific Helios-deficient cells enhanced antitumor activity in the TME, whereas systemic Helios-deficient Treg cells did not. Helios-deficient Treg cells increase IFN-*γ* and TNF-*α* expression, indicating phenotypic conversion. By contrast, TI-Treg cell activity is downregulated by IFN-*γ* produced by Teff cells in the TME. Nrp1-deficient Treg cells produce IFN-*γ* in the TME, with the resultant IFN-*γ* reducing the suppressive activity of Treg cells without losing Foxp3 expression, a phenomenon called “Treg cell fragility” [56].

TI-Treg cells show specific gene expression patterns. A recent study compared the gene expression profiles of breast cancer-infiltrating Treg cells with those of Treg cells in the peripheral blood and normal tissue [41]. The overall gene expression pattern of TI-Treg cells was closer to that of normal breast tissue-resident Treg cells than that of peripheral Treg cells, suggesting that the tissue surrounding the tumor is the major determinant of Treg cell gene expression. TI-Treg cells express a few distinct genes, including those encoding chemokine receptor CCR8 and type I interferons. A similar approach showed the upregulation on Tl-Tregs in human cancers of gene-encoding surface markers [57], including those encoding several immune checkpoint receptors, such as IL1R2, PD-L1, PD-L2, and CCR8. The levels of expression of some of these gene products, including LAYN, MAGEH1, and CCR8, were found to correlate with poor prognosis. Further elucidation and characterization of TI-Treg-specific genes will help in precisely targeting these cells, without compromising general Treg cell activity in other parts of the body.

### 4.2. Suppressive Mechanisms of TI-Treg Cells

Although many studies have assessed the mechanisms by which Treg cells suppress immune responses in general, less is known about the mechanisms by which these cells suppress antitumor immunity. In addition, Treg cells acquire distinct immunomodulatory mechanisms when residing in different peripheral tissues [58]. Therefore, understanding TI-Treg-specific suppressive mechanisms is critical in developing therapeutic strategies to treat cancers without affecting Treg functions in general.

In many types of human cancers, including hepatocellular carcinoma, pancreatic cancer, and ovarian cancer, TI-Treg cells suppress antitumor activity by secreting the anti-inflammatory cytokines TGF-*β* and IL-10 and by upregulating the expression of inhibitory immune checkpoint receptors, including CTLA-4, GITR, TIM-3, and ICOS [7, 29, 59–64]. CTLA-4 has a higher avidity to B7.1 and B7.2 on DCs than CD28 does, thereby preventing Teff cell activation. TIM-3, LAG-3, and PD-1 also inhibit Teff cells and CD8^+^ CTLs. TI-Treg cells induce the exhaustion of CTLs characterized by inefficient release of cytotoxic granules, low expression of effector cytokines, and expression of the coinhibitory receptors PD-1 and TIM-3 [65]. These results suggest that TI-Treg cells use mechanisms common to Treg cells in general, as well as preferentially involving immune inhibitory receptors [66].

IDO exerts an important immunosuppressive effect in tumors. Interactions between CTLA-4 and DCs can induce the expression of IDO, resulting in the production of the immunosuppressive metabolite kynurenine [67]. Kynurenine can support Treg cell differentiation but impairs T cell cytotoxic activity [68, 69]. IDO is expressed at high levels in tumors and other immunomodulatory cells, leading to increased kynurenine levels in the TME and possibly enhancing Treg cell activity [70].

TI-Treg cells show high expression of CD39, which converts ATP into AMP, and of CD73, which converts AMP to adenosine [66]. Adenosine is a powerful anti-inflammatory factor that inhibits the function of immune cells by binding to the adenosine receptor 2A (A_2A_R) on Teff cells and upregulates intracellular cAMP level [66]. Adenosine also potentiates the differentiation, proliferation, and suppressor activities of Treg cells and MDSCs [66].

## 5. Immunotherapy Targeting TI-Treg Cells

Because Treg cells suppress antitumor immunity mediated by CD8 and CD4 Teff cells, immunotherapy targeting Treg cell function in TME is being actively pursued. Methods to target Treg cells include depletion of Treg cells, blocking immune checkpoint receptors, recruitment of Treg cells, and treatment of cells with inhibitory cytokines [66, 71, 72].

### 5.1. Depleting Treg Cells

CD25 is a well-known Treg cell marker. Depleting Treg cells by targeting CD25 has yielded conflicting results. The anti-CD25 monoclonal antibody daclizumab was reported to have beneficial effects in patients with glioblastoma and breast cancer [73, 74] but was reported to have a marginal effect in metastatic melanoma [75]. Similarly, the IL-2-diphtheria toxin fusion protein denileukin diftitox was effective in patients with renal cell carcinoma (RCC) [76] but had an adverse effect in metastatic melanoma [77]. Possible reasons for these conflicting results are the effect of these drugs on Teff cells, the rapid repopulation by Treg cells upon drug withdrawal, or the nonrecognition of Treg cells by immune conjugates following treatment with denileukin diftitox. Recently, CD25 was found to be preferentially expressed in tumors in vivo [78]. The commonly used rat IgG1-depleting antibody PC-61 does not effectively deplete Treg cells in tumors, because it binds to inhibitory Fc*γ*RIIb. Treatment with Fc-optimized anti-CD25 antibody (i.e., the Fc region of PC-61 was replaced by murine IgG2a and *κ* constant region) resulted in the effective depletion of Treg cells and an increase in the Teff-to-Treg ratio, leading to tumor regression and increased survival [78].

### 5.2. Immune Checkpoint Inhibitors

CTLA-4 is an immune checkpoint receptor highly expressed in Treg cells. Immune checkpoint receptors are immune inhibitory receptors that are often highly expressed in the TME [79]. The rationale for using immune checkpoint inhibitors is to block inhibitory signals to Teff cells and restore their antitumor activity. Immune checkpoint inhibitors showed significant activity in clinical trials of patients with melanoma, nonsmall cell lung cancer (NSCLC), and RCC [80–83]. In recent years, four immune checkpoint inhibitors have been approved by the FDA for the treatment of metastatic melanoma, NSCLC, advanced RCC, and Hodgkin's lymphoma: monoclonal antibodies targeting CTLA-4 (ipilimumab and tremelimumab) and PD-1 (nivolumab and pembrolizumab).

Mechanistically, anti-CTLA-4 was first thought to prevent Treg cells from intercepting costimulatory signals from DCs, resulting in DC-induced Teff cell activation and proliferation. Ipilimumab and tremelimumab induce significant activation and expansion of Teff and CD8 T cells [84–88]. The effect of ipilimumab was recently substantiated by depleting Treg cells via antibody-dependent cell-mediated cytotoxicity (ADCC) [89]. However, tremelimumab, which does not have ADCC activity, had a similar therapeutic effect, suggesting that Treg depletion may not be the main mechanism of ipilimumab.

Another Treg-specific marker GITR is also a target for TI-Treg cells. Unlike in Treg cells, GITR acts as a costimulatory molecule in Teff cells, suggesting a beneficial effect in cancer therapy. In animal models, anti-GITR antibody induced antitumor activity by increasing Teff cells [90]. Combined treatment with anti-GITR and anti-CTLA-4 antibodies synergistically induced antitumor activity in human patients [91]. OX40, a member of the TNF receptor family, has a mechanism of action similar to that of GITR; that is, anti-OX40 antibody stimulates Teff cells but inhibits Treg cells. Anti-OX40 antibody enhanced CD8 T cell-mediated antitumor immunity in animal models of cancer [92]. Antibodies against GITR and OX40 are now in clinical trials [93].

Combining Treg cell depletion with immune checkpoint inhibitors resulted in a synergistic effect in an animal model of Claudin-low breast cancer, a subtype of triple-negative breast cancer [94]. Treg cell depletion and immune checkpoint inhibitors each had little effect on tumor growth, whereas their combination greatly reduced tumor burden [94].

### 5.3. Blocking Treg Cell Recruitment

Infiltration of Treg cells into tumors is a prerequisite for their activity. TI-Treg cells express a variety of chemokine receptors, including CCR4, CCR5, CCR6, CCR7, CCR10, CXCR3, and CXCR4, and migrate efficiently in response to tumor-derived chemokines [23, 95, 96].

CCR4 is preferentially expressed on TI-Treg cells rather than on Teff cells [25], with the CCL17/22-CCR4 axis playing an important role in lymphomas and in breast, lung, ovarian, gastric, and prostate cancers [23, 95, 96]. A monoclonal antibody targeting CCR4 has shown promising results, effectively depleting Treg cells, both in vitro and in clinical trials in human cancer patients [96, 97].

CXCR3^+^ Treg cells selectively accumulate in ovarian cancer and block the interactions between CXCR3 and its ligands CXCL9, CXCL10, and CXCL11, thereby suppressing tumor growth [98].

### 5.4. Blocking Inhibitory Cytokines

Because the TME is rich in immunosuppressive cytokines that strengthen the activity of TI-Treg cells, neutralizing these cytokines may reestablish effective antitumor immunity. Genetic ablation or blocking of IL-10 or TGF-*β* signaling results in tumor regression [99–102]. In addition, neutralization of IL-35 or Treg-specific deletion of IL-35 was found to enhance antitumor T cell responses and reduce tumor growth in various mouse tumor models [103]. Interestingly, IL-35 produced by Treg cells promoted the expression of several inhibitory receptors, including PD-1, TIM-3, and LAG-3, leading to T cell exhaustion. The higher numbers of IL-35-expressing Treg cells present in tumors than in spleen can be exploited for tumor-specific blockade of Treg cell function without affecting Treg function in general [103].

## 6. Conclusions and Perspective

In recent years, tumor immunotherapy has drawn much attention because of its specific targeting ability and reduced side effects. Targeting Treg cells in cancer treatment was hampered by a lack of knowledge of the properties of TI-Treg cells. Understanding the phenotypic and functional properties of Treg cells is essential to effectively and specifically target TI-Treg cells in cancer therapy without compromising immune homeostasis in general. Future studies should include a search for TI-Treg-specific genes in human cancers and elucidate their roles in tumor progression. Treg cells are heterogeneous, with different functional properties. Similarly, TI-Treg cells likely have distinct functional properties depending on their TME, as tumors have different environments. Treg cells may preferentially use limited suppressive mechanisms that best fit their environment. Studies of cancer-specific suppressive mechanisms, including causative factors, interactions with other cells in the TME, and their functional significance, are warranted.
